# Proposal of a New Composite Score (DAMADECO) to Simultaneously Evaluate Asthma and CRSwNP Severity in Comorbid Patients

**DOI:** 10.3390/jcm14030957

**Published:** 2025-02-02

**Authors:** Maria D’Amato, Patrizio Pasqualetti, Elena Cantone, Marco Caminati, Matteo Bonini, Fabiano Di Marco, Carlotta Pipolo, Veronica Seccia, Giovanni Sotgiu, Eugenio De Corso

**Affiliations:** 1 Respiratory Department, Monaldi Hospital AO Dei Colli, Federico II University, 80131 Naples, Italy; 2 Department of Public Health and Infectious Diseases, Sapienza University of Rome, 00185 Roma, Italy; 3 Department of Neuroscience, Reproductive and Dentistry Science, Federico II University of Naples, 80138 Naples, Italy; 4 Allergy Unit and Asthma Center, Department of Medicine, University of Verona, 37129 Verona, Italy; 5 National Heart and Lung Institute, Imperial College London, London SW7 2AZ, UK; 6 Department of Health Sciences, Università degli Studi di Milano, Pulmonology ward, ASST Papa Giovanni XXIII, 24127 Bergamo, Italy; 7 Otolaryngology Unit, ASST Santi Paolo e Carlo, Department of Health Sciences, University of Milan, 20122 Milan, Italy; 8 U.O. Otorhinolaryngology Unit, Pisa University Hospital, 56124 Pisa, Italy; 9 Clinical Epidemiology and Medical Statistics Unit, Department of Medical, Surgical and Experimental Sciences, University of Sassari, 07100 Sassari, Italy; 10 Fondazione Policlinico Universitario Agostino Gemelli IRCCS, 00168 Roma, Italy

**Keywords:** asthma, chronic rhinosinusitis with nasal polyps, composite score, multidisciplinary, global airway disease

## Abstract

**Background**: Asthma and chronic rhinosinusitis with nasal polyps (CRSwNP) are chronic respiratory conditions that frequently coexist. However, an integrated assessment tool for both conditions is currently lacking. This study aimed to develop and preliminarily evaluate a composite score capable of simultaneously assessing asthma and CRSwNP in comorbid patients. **Methods**: An expert panel comprising three pulmonologists, one allergist/clinical immunologist, and four ear, nose and throat (ENT) specialists developed a tool to capture asthma and CRSwNP severity. The tool (D’Amato-De Corso score, or DAMADECO score) incorporates eight parameters, four specific to asthma and four specific to CRSwNP, to assign individual scores for each condition. A composite score is then calculated to reflect the overall disease burden (ranging from −8: poor control and +8: optimal control). A retrospective pilot study was conducted to evaluate the tool. **Results**: The DAMADECO composite score was applied to 21 comorbid patients. The mean partial scores for asthma and CRSwNP were −1.57 and −1.67, respectively, with a mean total composite score of −3.24. A total of 13 out of 21 patients had uncontrolled domains in both diseases, while fewer patients had only uncontrolled domains in asthma (1/21) or CRSwNP (6/21). The DAMADECO score also allows researchers to track disease progression and monitor treatment effectiveness. **Conclusions**: The preliminary results suggest that the DAMADECO score is a promising tool for simultaneously assessing asthma and CRSwNP, addressing the unmet need for an integrated approach to comorbid respiratory diseases. Further validation studies are needed to validate the tool in larger patient populations.

## 1. Introduction

Asthma is a complex airway disorder that affects approximately 262 million people worldwide, with a prevalence of 27 million within Western Europe [1]. Chronic rhinosinusitis with nasal polyps (CRSwNP) is another respiratory disease impacting the upper airways, with an estimated prevalence between 1 and 4% found to be greater in men than in women [2,3,4]. Recent estimates report up to 44% of patients with comorbid asthma and CRSwNP across five European countries (France, Germany, Italy, Spain, and the United Kingdom) and 52% in the United States [5]. Even higher percentages have been found in other studies [6]. According to the Italian severe asthma registry (SANI), 43% of patients with severe asthma suffer from concomitant CRSwNP [7].

Severe asthma, which affects approximately 5–10% of patients with asthma [8,9], is characterized by bronchial chronic inflammation that causes impactful respiratory symptoms, frequent exacerbations, increased access to the emergency room and the use of oral corticosteroids (OCS), as well as poor response to inhaled therapies [10,11,12]. CRSwNP features the inflammation of the upper airways, e.g., the nose and paranasal sinuses, and it is associated with the remodeling of sinonasal mucosa with nasal polyps’ development. The main symptoms of CRSwNP include chronic nasal congestion, loss of smell, and rhinorrhea [13]. Severe asthma patients with CRSwNP experience poor asthma symptoms together with a higher exacerbation frequency, an increased need for systemic steroid trials and a worse quality of life (QoL). Similarly, poor control of CRSwNP has been observed in patients with comorbid asthma [14,15,16].

The coexistence of these inflammatory disorders in comorbid patients is receiving increasing attention; growing evidence indicates the presence of shared pathophysiological processes involving both upper and lower airways that promote the development of CRSwNP in asthma patients and the other way around [17]. In light of the close relationship between the two conditions, asthma and CRSwNP can be interpreted as a single disease, formally described in the theory of “united airway disease” (UAD), in which upper and lower airways are conceived as a single functional unit [18,19].

The multidisciplinary evaluation of comorbid patients conducted by pulmonologists, allergists/clinical immunologists and ear, nose, and throat (ENT) specialists has been broadly encouraged [20,21,22,23]. De Corso et al. recently emphasized that CRSwNP patients should be evaluated in a multidisciplinary fashion to detect the presence of asthma; likewise, the presence of CRSwNP should be always assessed in patients with moderate to severe asthma [20]. However, a coordinated and multidisciplinary global airway approach is still poorly implemented in clinical practice, with scarce or even absent multidisciplinary outpatient clinics where patients undergo a systematic routine assessment of both upper and lower airways [24]. As recently suggested by Caminati et al., an integrated assessment of upper and lower airways should be systematically applied [25]. A high number of tools and patient-reported outcomes (PROs) are available to assess different aspects of asthma and CRSwNP, such as QoL, disease control, and symptom severity. However, all the existing tools have been designed and validated to assess either CRSwNP or asthma separately; a review of the literature confirms the absence of tools that can evaluate both conditions simultaneously [25].

Starting from these considerations, an Italian multidisciplinary group composed of pulmonologists, allergists/clinical immunologists and ENT specialists discussed the development of a novel tool specifically designed for comorbid patients. The expert authors met to analyze the gaps and needs in assessing comorbid patients in clinical practice, for which the tool may provide support. They defined the tool’s objectives and expected outcomes and determined the relevance of its use. The authors also proposed the domains and parameters the tool should investigate to capture the severity of asthma and CRSwNP effectively. A preliminary analysis was conducted on a restricted cohort of 21 comorbid patients, whose data were retrospectively collected. This exploratory study aimed to ascertain whether the tool can distinguish the impact of the two diseases separately while also providing insights into the overall burden of these diseases as a collective entity.

## 2. Material and Methods

### 2.1. Definition of the Tool’s Objectives and Expected Outcomes

Given the need for enhanced multidisciplinary evaluation of comorbid patients and the lack of suitable tools, a panel composed of three pulmonologists, one allergist/clinical immunologist and four ENT specialists with proven expertise in managing comorbid patients and prescribing biological therapies agreed that a composite score for the simultaneous assessment of asthma and CRSwNP would be highly desirable. The panel agreed that the tool should aim to simultaneously track the severity of each condition over time, assisting clinicians in optimizing diagnostic evaluations and treatment strategies in clinical practice.

The authors of this paper further agreed that the tool should provide two separate scores to measure the severity of each pathology independently, as well as a composite score to determine the overall burden of asthma and CRSwNP, intended as a global airway disease. The experts recommended the tool to be used by all the specialists involved in the management of asthma patients, in particular, pulmonologists, allergists/clinical immunologists and ENT specialists.

The board recognized the potential of the tool to facilitate treatment decisions, maximize the treatment outcomes of both diseases and encourage an integrated multidisciplinary management of comorbid patients.

### 2.2. Definition of Strategic Parameters for Both Asthma and CRSwNP

The experts identified strategic evaluations to assess symptoms, function, and systemic steroid use to capture the impact of the two conditions. Among these, clinically relevant parameters and validated tools were selected, prioritizing those recommended by current guidelines and/or widely implemented in routine practice.

The parameters selected by the board are summarized in Table 1. Asthma parameters (exacerbations, pre-bronchodilator forced expiratory volume in the 1st second, predicted [pre-BD FEV_1_ pred.], asthma control test [ACT] score, and OCS use) were chosen according to the pivotal European Respiratory Society (ERS)/American Thoracic Association (ATS) definition of severe and uncontrolled asthma [8]. The significance of these metrics in describing overall asthma control is also acknowledged by the Global Initiative for Asthma (GINA) recommendations [26]; furthermore, the same variables have been used to define asthma clinical remission [27]. It should be specified that asthma exacerbations may be of different severity, with the most serious requiring treatment with systemic corticosteroids, hospitalization, and mechanical ventilation [8].

Similarly, the metrics selected to evaluate the severity of CRSwNP (visual analogue scale [VAS] olfactory, nasal polyp score [NPS], sino-nasal outcome test-22 [SNOT-22], OCS use) were used by the recent European Position paper on Rhinosinusitis and Nasal Polyps (EPOS) and European Forum for Research and Education in Allergy and Airway disease (EUFOREA) to define CRSwNP state, overall disease control and remission [28].

Regarding OCS use, the experts agreed that the number of OCS cycles is easier to retrieve as compared with other variables (i.e., cumulative OCS dosage or the total days of OCS). While continuous (maintenance) OCS use is often necessary for managing both asthma and CRSwNP and represents the highest level of OCS dependence, its temporary use may provide a more precise measure for determining the level of dependence in both diseases. 

### 2.3. Cutoff Values of Asthma and CRSwNP Parameters and Proposal of a New Scoring System (DAMADECO Score)

Cutoff values for each parameter were determined according to international recommendations and/or consensus statements, enabling the classification of patients into three categories that define the control of asthma and CRSwNP as either inadequate, intermediate, or adequate. When evidence from the literature was limited or ambiguous, the proposed thresholds were established based on the collective clinical expertise of the panel members, particularly regarding the definition of cutoff values for intermediate control of the two diseases.

Briefly, inadequate asthma control was defined as follows: (1) ≥2 exacerbations during the previous year [8]; (2) pre-BD FEV_1_ pred. < 70%; (3) ACT score < 20 [26]; (4) ≥2 OCS cycles during the previous year for treating exacerbations [8]. The definition of inadequate CRSwNP relied on the following factors: (1) a VAS olfactory score ≥ 7 [29], (2) NPS score > 4 [30,31], (3) SNOT-22 score ≥ 40 [32], (4) ≥2 OCS cycles administered during the previous year. Although continuous (maintenance) OCS use was not explicitly included in the domain, the authors agreed that patients using maintenance OCS would be classified as having “inadequate” OCS dependence, similar to patients who required at least 2 OCS courses in the previous year.

These categories were scored as follows: inadequate = −1, intermediate = 0, adequate = 1. The sum of the scores obtained for each domain indicates the severity of asthma and CRSwNP as separate conditions, with higher scores denoting better control of each disease. The sum of asthma and CRSwNP individual scores informs on the overall impact of these disorders as a unified global airway disease, with the worst clinical scenario corresponding to a composite score value of −8, while the best corresponds to +8. Table 2 reports the cutoff values that define the three control categories for each of the disease domains, and the scoring associated with each of the categories.

### 2.4. Patient Population

A pilot study was conducted to test the DAMADECO score. Data from 21 comorbid patients were retrospectively collected. Of those, 10 patients were followed by a pulmonary unit (Ospedale “Vincenzo Monaldi”, AOS dei Colli, Napoli, Italy) and 11 patients were followed by an ENT unit (Policlinico Universitario Agostino Gemelli IRCCS, Roma, Italy). Demographic and clinical characteristics were collected, including age, sex, body mass index (BMI), total immunoglobulin (Ig)E levels, blood eosinophil count, and non-steroidal anti-inflammatory drug-exacerbated respiratory disease (NSAID-ERD). Asthma-specific parameters included asthma duration, fractional exhaled nitric oxide (FeNO), number of exacerbations in the previous year, number of asthma emergency room accesses in the previous year, OCS maintenance use, number of OCS cycles, pre-BD FEV_1_ (absolute and pred.), and ACT score. CRSwNP-specific parameters included number of OCS cycles, VAS olfaction, VAS nasal obstruction, VAS rhinorrhea, nasal congestion score (NCS), Sniffin sticks identification test, Lund Mackay score, SNOT-22 and NPS. Informed consent was obtained from all patients; this study was conducted in conformity with the Declaration of Helsinki and was approved by the Ethics Committee of Vanvitelli University—AO Dei Colli (AOC-0010488-2024, 13 June 2024).

### 2.5. Statistical Analyses

Descriptive statistics (mean and standard deviation [SD], median and range, proportions) were used to characterize patients according to type/distribution of corresponding data. Since scores were measured at the ordinal level, Spearman’s rank rho was computed to assess their correlation.

Only those patients with a predicted percentage of missing values < 10% were selected. SPSS 27.0 (IBM) was used as the statistical software.

## 3. Results

Data were collected from 21 patients with asthma and CRSwNP. Table 3 shows the patients’ demographic and clinical characteristics. There was a predominance of female participants (n = 14, 67%); on average, the patients were overweight, with a BMI of 26.1 kg/m^2^. The patients also presented an elevated median level of total immunoglobulin E (IgE) (270 UI/L, range 38–2198) and a high median count of blood eosinophils (460 cells/mm^3^, range 30–1670); a total of 4 patients out of 20 had NSAID-ERD. Median asthma duration was 25 years (range 1–54); the patients had a median of 3 (range 0–8) asthma exacerbations in the previous year and had a suboptimal median pre-bronchodilator forced expiratory volume in one second (pre-BD FEV_1_ pred. = 69%). Accordingly, the ACT score reflected a poor control of the disease (median ACT score = 14, range 6–24). The majority of patients (n = 13, 62%) used OCS in a continuous manner to treat asthma; the rest of the patients took a median of 3 (range 0–10) OCS cycles per year. The patients took a median of 1 (range 0–7) OCS cycles per year to treat CRSwNP. The median values of VAS olfaction (7, range 2–10), VAS nasal obstruction (6, range 1–8), VAS rhinorrea (4, range 0–9), NCS (2, range 1–4), Sniffin sticks identification test (10, range 0–16), Lund Mackay score (16, range 2–24), SNOT-22 (59, range 16–83) and NPS (4, range 0–8) indicated the severity of CRSwNP.

The DAMADECO composite score described above was applied to the pilot sample. The mean partial score obtained for asthma was −1.57 (SD = 2.27), while the mean partial score for CRSwNP was −1.67 (SD = 1.28) and the mean total composite asthma–CRSwNP score was −3.24 (SD = 2.72).

Overall, 10 out of the 21 patients (47%) showed a worse partial composite score for asthma compared to CRSwNP, whereas 9/21 (43%) showed a worse score for CRSwNP compared to the partial asthma score (two ties were observed).

**Figure 1:**
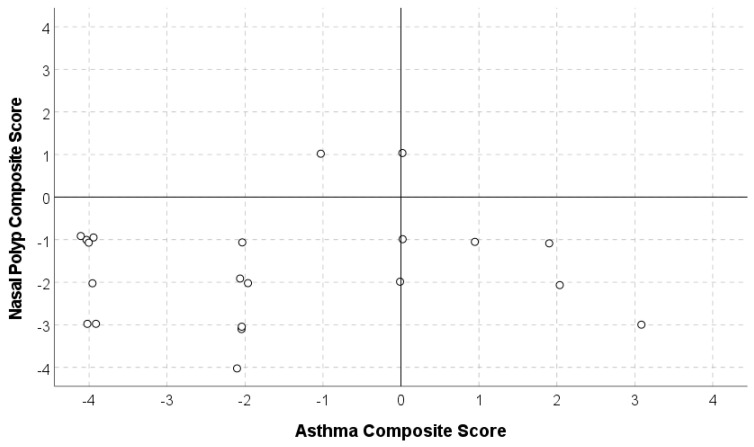
Scatter plots illustrating asthma and CRSwNP nasal composite score. Variables were plotted for each asthma/CRSwNP partial scores (asthma domains expressed in the X axis; CRSwNP expressed in the Y axis).

Figure 1 reports the scatter plot illustrating the distribution of asthma and CRSwNP nasal composite scores. Spearman’s rank correlation resulted in rho = −0.011, a substantially null correlation (*p* = 0.961). In general, dots located in the bottom-left quadrant represent patients who have a higher number of uncontrolled domains compared to controlled domains in both pathologies (13/21). Dots in the top-left quadrant indicate patients with a predominance of uncontrolled domains in asthma (1/21). Dots in the bottom-right quadrant indicate patients with uncontrolled domains predominantly in CRSwNP (6/21). Only one patient obtained a score = 0 in asthma (suggesting that the patient had a partial control in all asthma domains or a balancing between uncontrolled and controlled asthma domains) and a score of 1 in CRSwNP. The scatter plot also shows that while some comorbid patients may have one disease that is more severe compared to the other, there are also cases where both asthma and CRSwNP are simultaneously severe. The very small correlation between the asthma and CRSwNP scores indicates that the asthma score alone does not inform about CRSwNP clinical status. Thus, these data preliminarily reinforce the need for a composite asthma and CRSwNP assessment tool specific for comorbid patients.

**Figure 2:**
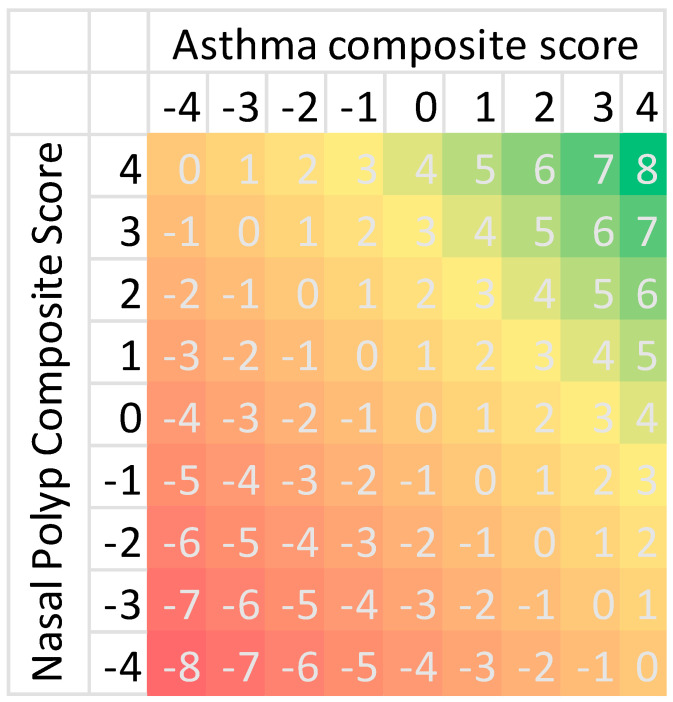
Composite scores for asthma and CRSwNP. The grid illustrates all possible combinations of asthma and CRSwNP composite scores derived from the DAMADECO tool. Each point on the grid represents a unique composite score resulting from the sum of individual asthma and CRSwNP scores. The color coding visually indicates the range of disease severity outcomes, with red representing the maximum severity for both diseases and green representing the maximum control for both diseases.

Figure 2 illustrates the full spectrum of possible composite scores resulting from the sum of individual asthma and CRSwNP scores (from −8 to +8). The colorimetric gradient (obtained by means of “conditional formatting” option in Excel, Microsoft 365) visually indicates the degree of disease control, with red representing the maximum severity for both diseases and green representing the maximum control for both diseases. This approach allows for quick interpretation and provides insight into the clinical significance of each score, though further studies are necessary to establish precise cutoff values for clinical interpretation. Ideally, a patient with full control over both pathologies should achieve a score of 8 (green), while a score below 2 (orange-red) may suggest a severe condition.

**Figure 3:**
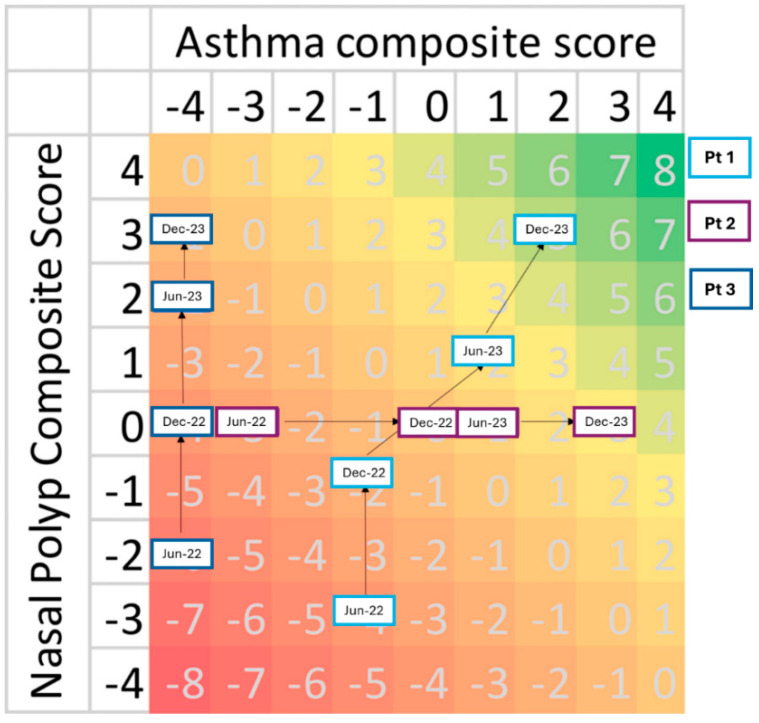
Applicability of the DAMADECO score: tracking disease progression and treatment response. This figure illustrates the possibility to monitor changes in asthma and CRSwNP severity over time using the DAMADECO tool. The baseline scores of three fictional cases (Pt1, Pt2 and Pt3) and the improvement in their scores during follow-ups are represented in the grid. The color coding visually indicates the range of disease severity outcomes, with red representing the maximum severity for both diseases and green representing the maximum control for both diseases.

Figure 3 exemplifies the applicability of the DAMADECO score in monitoring changes in asthma and CRSwNP severity over time in three fictional cases (Pt1, Pt2, Pt3). The composite scores of Pt1, Pt2, Pt3 are shown at baseline (June 2022) and at three follow-up visits, with the last visit taking place in December 2023. A detailed explanation of score changes and their clinical implications is provided below for each fictional case.

(1) Pt1 initially shows severe asthma and CRSwNP, as indicated by asthma and CRSwNP scores, both being lower than 0 at baseline (asthma score = −1, CRSwNP score = −3), and corresponding to a composite score of −4. Thus, the best approach for this patient would be prescribing an effective therapy that can treat both diseases to improve both partial scores and, consequently, the overall composite score. By December 2023, Pt1 demonstrates significant improvement in both conditions (asthma score = +2; CRSwNP score = +3), reaching a composite score of +5 (green area). The composite and partial scores demonstrate the therapeutic success of the chosen treatment in improving both diseases.
(2) Pt2 is severely affected by asthma at baseline (asthma score = −3), while CRSwNP has no impact on the composite score (CRSwNP score = 0). In this case, the clinician should prioritize a therapeutic approach that focuses on alleviating asthma symptoms rather than treating CRSwNP. By December 2023, Pt2 shows notable improvement in asthma symptoms (asthma score = +3) while maintaining an unchanged CRSwNP score, reaching a composite score of +3. The score changes confirm the beneficial effects achieved with the chosen anti-asthma therapy.
(3) Pt3 shows severe symptoms in both asthma and CRSwNP at baseline (asthma score = −4; CRSwNP score = −2, composite score = −6). Like Pt1, Pt3 would also benefit from a highly effective therapy to treat both asthma and CRSwNP. However, differently from Pt1, this patient appears to improve CRSwNP outcomes only (CRSwNP score = +3), without any improvement in asthma, whose score remains −4 until the last follow-up. By December 2023, Pt3’s composite score is −1. Overall, the partial and composite scores indicate that the treatment effectively reduced CRSwNP severity but failed to improve asthma control.

By tracking the changes in asthma and CRSwNP scores on the grid, clinicians can assess the effectiveness of treatment interventions and adjust care plans as needed.

## 4. Discussion

The pathophysiological process underlying asthma and CRSwNP is mostly driven by shared inflammatory mechanisms, which involve a complex activation of innate and adaptive immune cells, and increased production of several pro-inflammatory cytokines (e.g., interleukin (IL)-4, IL-5, IL-13, thymic stromal lymphopoietin (TSLP)). Such an inflammatory environment is usually associated with an eosinophilic endotype, with or without concomitant allergies, as signs of a type-2-skewed inflammatory response [33]. Based on the common inflammatory milieu, both severe asthma and CRSwNP symptoms can be treated by inhaled corticosteroids but frequently require OCS if symptoms remain uncontrolled; as a consequence, comorbid patients often receive OCS and have a higher risk of developing OCS-related adverse events [14].

The advent of biologics has expanded the variety of therapeutic options to manage comorbid patients with uncontrolled symptoms and has allowed for the possibility to target asthma and CRSwNP with shared inflammatory pathways. Several monoclonal antibodies (mAbs) targeting distinct type 2 inflammatory players (omalizumab, mepolizumab, reslizumab, benralizumab, dupilumab, and tezepelumab targeting IgE, IL-5, IL-5 receptor, IL-4 receptor, and TSLP, respectively), initially available for the treatment of severe asthma only, were found to significantly reduce CRSwNP inflammation and symptoms when used to treat comorbid patients. Among them, omalizumab, dupilumab, and mepolizumab have already been licensed for the treatment of patients with CRSwNP even in the absence of asthma [24,33].

Despite the huge therapeutic potential offered by biologics for the treatment of asthma and CRSwNP, there is still large variability in the extent of the response to biologics in both CRSwNP and asthma outcomes [24], and a not-trivial percentage of comorbid patients do not achieve adequate control of both conditions with the initial biologic treatment. In case of a suboptimal response for either asthma or CRSwNP, patients may need to switch to a different biologic therapy [34].

To improve the understanding of these conditions, optimize the care of comorbid patients and increase the treatment success rate, asthma and CRSwNP need to be recognized, assessed and treated as a single airways disease, already known as UAD. A change in the management of comorbid patients towards a more comprehensive approach has been already warranted [35] and constructive recommendations have been formulated to facilitate clinicians’ everyday practice by merging asthma- and CRSwNP-specific guidelines [23]. While the establishment of a unique integrated care pathway may be challenging due to the heterogeneous manifestation of patients with asthma and CRSwNP, Seccia et al. identified three patient profiles (patient with asthma complaining about nasal symptoms, patient with severe asthma receiving biologic treatment but complaining about nasal symptoms, patient with CRSwNP complaining about asthma symptoms) and suggested three distinct tracks to support the decision-making process when dealing with similar cases [21]. Importantly, Backer and colleagues highlighted the need for further research to advance the screening, diagnosis, and choice of treatment in patients with coexisting asthma and CRSwNP. Among various aspects to be explored, the need to generate a composite tool to characterize comorbid patients and guide their management has been highlighted by different authors [23,25].

In this manuscript, the panel composed by pulmonologists, allergists/clinical immunologists and ENT specialists endorsed the development of a composite score for the simultaneous assessment of asthma and CRSwNP in comorbid patients to support clinicians in their routine clinical practice. The experts set the objectives of the score and outlined the domains and parameters to be included. The severity of asthma and CRSwNP will be quantified by two separate scores obtained from the assessment of asthma and CRSwNP-specific metrics; the combination of the two separate scores will inform on the overall severity of asthma and CRSwNP as a global airway disease. Importantly, all the parameters included in the score are already used to assess asthma and CRSwNP in routine clinical practice.

The preliminary analysis conducted on a restricted number of comorbid patients confirmed that the tool can discriminate the severity of each condition as well as inform on the overall asthma–CRSwNP impact for each of the defined domains.

The experts believe that tools like this may have the potential to improve clinical practice by providing a comprehensive, streamlined approach to optimize the multidisciplinary management of comorbid patients, ultimately improving their clinical outcomes and well-being. Nevertheless, a prospective, multicenter study with a larger patient population should be conducted to fully validate this tool by confirming its validity and reliability, and establish cutoff points for clinical decision-making.

From this preliminary analysis, indexes of central tendency and dispersion, although based on a small sample, may allow us to plan a prospective study and estimate the appropriate sample size.

## Figures and Tables

**Table 1:** Asthma- and CRSwNP-specific parameters that define the domains included in the composite score.

Asthma	CRSwNP
Exacerbations (N, during the previous year)	VAS olfaction (most recent assessment)
Pre-BD FEV_1_ pred. (most recent assessment)	NPS (most recent assessment)
ACT (most recent assessment)	SNOT-22 (most recent assessment)
OCS cycles (N, during the previous year)	OCS cycles (N, during the previous year)

ACT: asthma control test; CRSwNP: chronic rhinosinusitis with nasal polyps; NPS: nasal polyp score; OCS: oral corticosteroids; Pre-BD FEV_1_, pred.: forced expiratory volume in 1 s, predicted; SNOT-22: sino-nasal outcome test-22; VAS: visual analogue scale.

**Table 2:** Cutoff values defining asthma and CRSwNP control categories and proposed scores. In the right column, the total asthma score is calculated by summing the individual asthma item scores, while the total CRSwNP score is obtained by adding up the individual CRSwNP item scores.

Asthma	ADEQUATE (1)	INTERMEDIATE (0)	INADEQUATE (−1)	Item Scores
OCS cycles (N)	0	1	≥2	
ACT (score)	≥25	≥20, <25	<20	
Exacerbations (N)	0	1	≥2	
Pre-BD FEV_1_ (% predicted)	≥80	≥70, <80	<70	
				Total Asthma Score
CRSwNP	ADEQUATE (1)	INTERMEDIATE (0)	INADEQUATE (−1)	Item scores
OCS cycles (N)	0	1	≥2	
SNOT-22	<20	≥20, <40	≥40	
VAS olfactory (score)	≤3	>3, <7	≥7	
NPS	≤2	>2, ≤4	>4	
				Total CRSWNP Score

ACT: asthma control test; CRSwNP: chronic rhinosinusitis with nasal polyps; NPS: nasal polyp score; OCS: oral corticosteroids; Pre-BD FEV_1_: pre-bronchodilator forced expiratory volume in 1 s, predicted; SNOT-22: sino-nasal outcome test-22; VAS: visual analogue scale.

**Table 3:** Patients’ baseline characteristics. Data are from 21 patients unless otherwise specified.

GENERAL CHARACTERISTICS		
Age, years (mean, SD)	53.7	11.6
Female (n, %)	14	67%
BMI, kg/m^2^ (mean, SD)	26.1	3.3
Total IgE, UI/L (median, range)	270	38–2198
Blood eosinophil count, cells/mm^3^ (median, range)	460	30–1670
NSAID-ERD (n, %) (data from n = 20 patients)	4	20%
ASTHMA PARAMETERS		
Asthma duration, years (median, range)	25	1–54
FeNO, ppb (median, range)	31.5	8–128
Number of exacerbations in the previous year (median, range)	3	0–8
Number of asthma emergency room accesses in the previous year (median, range)	0	0–4
Patients with asthma continuative OCS use (n, %)	13	62%
Number of brief OCS cycles (median, range)	3	0–10
Pre-BD FEV_1_ pred., % (median, range)	69	29–98
Pre-BD FEV_1_, absolute (L) (median, range)	1.8	0.6–3.1
ACT score (median, range)	14	6–24
CRSWNP PARAMETERS		
Number of brief OCS cycles for nasal polyps (median, range)	1	0–7
VAS olfaction (median, range)	7	2–10
VAS nasal obstruction (median, range)	6	1–8
VAS rhinorrhea (median, range)	4	0–9
NCS (median, range)	2	1–4
Sniffin sticks identification test (median, range)	10	0–16
Lund Mackay score (median, range)	16	2–24
SNOT-22 (median, range)	59	16–83
NPS (median, range)	4	0–8

ACT: asthma control score; BMI: body mass index; CRSwNP: chronic rhinosinusitis with nasal polyps; FeNO fractional exhaled nitric oxide; Ig: Immunoglobulin; NCS: nasal congestion score; NPS: nasal polyp score; NSAID-ERD: non-steroidal anti-inflammatory drug-exacerbated respiratory disease; OCS: oral corticosteroids; Pre-BD FEV_1_.: pre-bronchodilator forced expiratory volume in 1 s; SNOT-22 sino-nasal outcome test-22; VAS: visual analogue scale.

## Data Availability

The datasets presented in this study are not readily available because they will be used for future validation studies. Requests to access the datasets should be directed to the corresponding author.
